# A disproportionate contribution of papillary muscles and trabeculations to total left ventricular mass makes choice of cardiovascular magnetic resonance analysis technique critical in Fabry disease

**DOI:** 10.1186/s12968-015-0114-4

**Published:** 2015-02-21

**Authors:** Rebecca Kozor, Fraser Callaghan, Michel Tchan, Christian Hamilton-Craig, Gemma A Figtree, Stuart M Grieve

**Affiliations:** North Shore Heart Research Group, Kolling Institute of Medical Research, University of Sydney, Sydney, Australia; Department of Cardiology, Royal North Shore Hospital, Sydney, Australia; Sydney Translational Imaging Laboratory, Sydney Medical School, University of Sydney, Sydney, Australia; Department of Genetic Medicine, Westmead Hospital, Sydney, Australia; Discipline of Genetic Medicine, Sydney Medical School, University of Sydney, Sydney, Australia; Heart and Lung Institute, The Prince Charles Hospital, Brisbane, Australia; University of Queensland, Brisbane, Australia

**Keywords:** Fabry disease, Papillary muscles, Left ventricular hypertrophy, Cardiovascular magnetic resonance imaging

## Abstract

**Background:**

Sphingolipid deposition in Fabry disease causes left ventricular (LV) hypertrophy, of which the accurate assessment is essential. Cardiovascular magnetic resonance (CMR) has been proposed as the gold standard. However, there is debate in the literature as to whether papillary muscles and trabeculations (P&T) should be included in LV mass (LVM).

**Methods/results:**

We examined the accuracy of 2 CMR methods of assessing LVM and LV volumes, including (M_*inc*_P&T) or excluding (M_*ex*_P&T) P&T, in a cohort of Fabry disease subjects (n = 20) compared to a matched control group (n = 20). Significant differences between the two measurement methods were observed for LV end-diastolic volume, LV end-systolic volume, LVM, and LV ejection fraction (LVEF) in both groups. These differences were significantly greater in the Fabry group compared to controls, except for LVEF. P&T contributed to a greater percentage of LVM in Fabry subjects than controls (20 ± 1% vs 13 ± 2%, p = 0.01). In the control group, both volume-derived methods (M_*inc*_P&T or M_ex_P&T) provided accurate SV measurements compared with the internal reference of velocity-encoded aortic flow. In the Fabry group, inclusion of P&T (M_*inc*_P&T) resulted in good concordance with phase contrast flow imaging (difference between flow and volume techniques: 1 ± 3 ml, p = 0.7).

**Conclusion:**

The volumetric contribution of P&T in Fabry disease is markedly increased relative to healthy controls. Failure to account for this results in significant underestimation of LVM and results in misclassification of a proportion of subjects.

## Background

**Figure 1:**
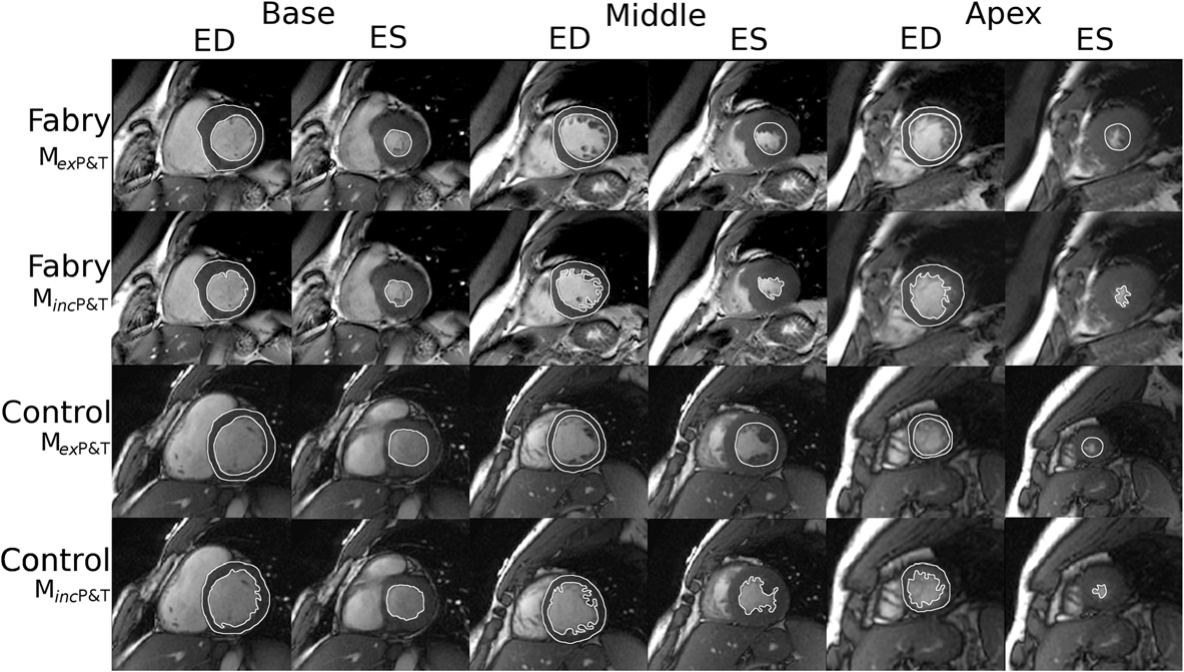
**Illustrative example of ventricular assessment.** The top two rows show data from an example Fabry subject, and the bottom two show data from an example control subject. For each cohort, the top row panels show epicardial and endocardial contours drawn using Method 2 (M_ex_P&T) during end-diastole (columns labelled ‘ED’) and endocardial contours during end-systole (‘ES’ columns) for representative short axis views at base, middle and apex levels (‘Base’, ‘Middle’ and ‘Apex’ headings). The bottom row for each example show the corresponding contours drawn using Method 1 (M_inc_P&T), which includes the trabeculations and papillary muscles.

Fabry disease is a X-linked disorder characterized by deficient activity of α-galactosidase A, which leads to progressive lysosomal accumulation of complex sphingolipids, predominantly globotriaosylceramide [1]. In the myocardium this typically produces a uniform pattern of left ventricular hypertrophy (LVH) that involves the papillary muscles and trabeculations (P&T), as well as the ventricular walls. Cardiac death is a major contributor to mortality in Fabry disease, and occurs most commonly secondary to arrhythmias and heart failure. LVH and hypertension are the factors that are most associated with cardiac death in Fabry patients [2]. Serial monitoring of left ventricular function (LVF) and left ventricular mass (LVM) in these patients is therefore desirable for both monitoring the progression of disease, and for assessing the response to treatments such as enzyme replacement therapy (ERT). Cardiovascular magnetic resonance (CMR) has been proposed as the ‘gold standard’ non-invasive method of measuring these indices, however, there is debate in the literature as to whether P&T should be included in the cavity volume or the myocardial mass [3-6]. Figure 1 illustrates these two approaches to ventricular quantification. The Society of Cardiovascular Magnetic Resonance task force on standardized protocols does not currently favor one method over the other, but suggests that inclusion or exclusion of papillary muscles in LVM should be the same as that used in normal reference ranges used for comparison [7].

In this study, we compare two different CMR approaches in quantifying left ventricular volumes, function and mass in a cohort of Fabry disease subjects compared to controls. We hypothesized that incorporating the P&T during measurements of LVM is of greater importance in patients with ventricular hypertrophy.

## Methods

### Study population

Twenty subjects with known Fabry disease (genotype positive), from the New South Wales and Queensland states Fabry registry databases, who had been referred for CMR as part of clinical management were included. To avoid any influence of gender on our analysis, only male patients were included. Twenty age-matched male controls were randomly selected from a database of ‘normal’ healthy volunteers with no history of cardiac disease. The study was approved by the relevant Institutional Human Ethics Committees.

### Cardiovascular magnetic resonance imaging

MRI data was acquired using Balanced Steady-State Free Precession (bSSFP) cine imaging on a 1.5 T Tesla Signa HDxt GE system (TE 1.5 ms; TR 3.4 ms; 20 phases; flip angle 45°; acquisition matrix 224 × 224; FOV 35 cm; slice thickness 8 mm; slice gap 2 mm) or a 3.0 Tesla GE system (TE: 1.1-1.6 ms; TR: 3.1-3.6 ms; 20 phases; flip angle 40-45°; matrix 256 × 256; in-plane resolution 1.4 mm; slice thickness 8 mm; no inter-slice gap). Cardiac chamber volumes and myocardial mass were quantified using a short axis stack of images acquired during end-expiratory breath hold. Phase contrast velocity encoded data was acquired in the ascending aorta at the level of the main pulmonary artery bifurcation using breath-held gradient echo flow-encoded cine images acquired with 20 phases (TE: 3.8 ms; TR: 7.0-8.3 ms; matrix 256 × 256; in-plane resolution 1.4 mm; slice thickness of 8 mm).

### Image analysis

CMR images were viewed using Osirix software (http://www.osirix-viewer.com) [8]. Analysis was performed using the STIL analysis tool plugin for Osirix (http://www.stil.net.au/downloads). LV end-diastolic and end-systolic volumes, LV mass (LVM), LV stroke volume (SV) and LV ejection fraction (LVEF) were obtained from the short axis stack by manually contouring end-diastolic and end-systolic endocardial borders and end-diastolic epicardial borders from the base to the apex. The 4-chamber and LVLA views were used to confirm the segmentation to ensure accuracy at the base [9]. The LV endocardial border was defined using the two methods described below and depicted in Figure 1:

- M_*inc*_P&T – Papillary muscles and trabeculations with signal intensity within 1 standard deviation of the myocardial signal measured in the LV free wall were manually outlined and included in the myocardial area. Trabeculations below 1.5 mm were also not included in the segmentation [6].
- M_ex_P&T-–Papillary muscles and trabeculations were excluded from the myocardial area and included in the blood pool by defining the endocardial border as a continuous contour following a smooth path along the compacted myocardium, not including the distinct trabeculations and papillary muscles.

All endocardial and epicardial borders were contoured manually by both an experienced CMR Radiologist (SMG) and Cardiologist (RK). Both endocardial and epicardial borders were traced at end-diastole and only the endocardial borders at end-systole. End-diastole was defined visually as the phase with the largest intracavity volume, and end-systole as the phase with the smallest intracavity volume. The basal slice was selected when at least fifty percent of the left ventricular cavity was surrounded by myocardium at end-diastole. The apical slice was selected as the last frame showing intracavity blood pool at end-diastole. LV volumes and mass were compared between the 2 methods described above in both Fabry and control subjects. As an internal reference, SV calculated using the two methods was then compared with aortic SV measured by velocity-encoded flow sequences in the ascending aorta. Many sites that employ a M_ex_P&T approach match the LVM calculated from end-systole with the end-diastolic LVM (as a form of internal reference to ensure the same volume of P&T muscle was included in the blood pool at both end-systole and end-disatole). We chose not to perform this step, as this was felt unnecessary for the purposes of our comparison. Aortic outflow was calculated off-line and manually on a workstation (ReportCARD, GE Healthcare, Milwaukee, WI).

To verify the reproducibility and reliability of the different methods, the intra-observer and inter-observer repeatability of LVEDV and LVESV measurements were assessed in half of the subjects. For intra-observer variability, the measurements were performed twice by the same observer (RK) with at least 1 week between measurements. For inter-observer variability, the first measurements from observer 1 were compared to the measurements calculated by a second independent observer (SG), who was blinded to the initial results.

### Statistical analysis

Statistical analyses were carried out using SPSS 21 (IBM, Armonk, NY). All continuous variables are expressed as mean ± standard error of the mean. Categorical variables are expressed as frequencies or percentages. Outcome variables were compared using paired-samples t-test for matched variables within each subject group, and independent-samples t-test for variables between the groups. A p-value of <0.05 was considered statistically significant. The Bland Altman statistical test was used to assess intra- and inter-observer variability, with the results presented graphically including mean differences and 95% limits of agreement, and the coefficient of repeatability.

## Results

### Participant characteristics

All participants were males. The Fabry and control groups had a mean age of 42 ± 3 vs 35 ± 1 years (p = 0.08), and range 13–66 vs 27–56 years, respectively.

### Cardiovascular measures

**Figure 2:**
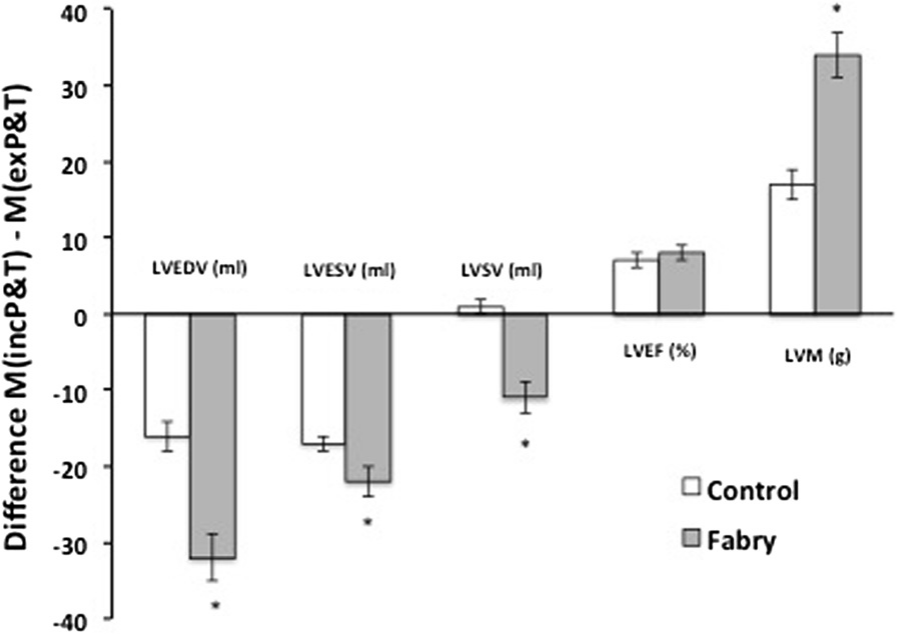
**Mean difference between measurement approaches** **(M**_***inc***_ **P&T** - **M**_***ex***_ **P&T** **)** **for key left ventricular measures of function and mass**, **in control and Fabry subjects.** LVEDV = left ventricular end diastolic volume, LVESV = left ventricular end systolic volume, LVSV = left ventricular stroke volume, LVEF = left ventricular ejection fraction, LVM = left ventricular mass.

The measures of LV volumes and mass obtained using the two methods (M_*inc*_P&T and M_*ex*_P&T) are summarized in Table 1. The two measurement methods resulted in significantly different LV end-diastolic volume (LVEDV), LV end-systolic volume (LVESV), LVM (measured in diastole) and LVEF for both groups. However, the choice of method made a more significant difference in the Fabry population. As shown in Figure 2, the differences between M_*inc*_P&T and M_*ex*_P&T methods were markedly greater in the Fabry group compared to the controls for LVEDV, LVESV and LVM.

**Table 1:** **Comparison of differences between methods** (**M**_***inc***_ **P&T** **and M**_***ex***_ **P&T)** **in control and Fabry subjects**

		**CONTROL SUBJECTS** **(n** **=** **20)**			**FABRY SUBJECTS** **(n** **=** **20)**	
	**M** **(incP&T)**	**M** **(exP&T)**	**p-** **value**	**M** **(incP&T)**	**M** **(exP&T)**	**p-** **value**
**LVEDV** **(ml)**	163 ± 6	179 ± 6	<0.001	127 ± 7	160 ± 8	<0.001
**LVESV** **(ml)**	54 ± 4	71 ± 3	<0.001	34 ± 2	56 ± 3	<0.001
**LVSV** **(ml)**	108 ± 5	105 ± 5	0.2	103 ± 7	100 ± 8	0.3
**LVEF** **(%)**	67 ± 1	61 ± 1	<0.001	73 ± 1	65 ± 1	<0.001
**LVM** **(g)**	130 ± 6	113 ± 6	<0.001	178 ± 14	144 ± 12	<0.001
**LVM indexed** **(g/** **m2)**	66 ± 3	58 ± 3	<0.001	98 ± 8	79 ± 7	<0.001

*LVEDV* = left ventricular end-diastolic volume, *LVESV* = left ventricular end-systolic volume, *LVSV* = left ventricular stroke volume, *LVEF* = left ventricular ejection fraction, *LVM* = left ventricular mass, *SV* = stroke volume.

**Figure 3:**
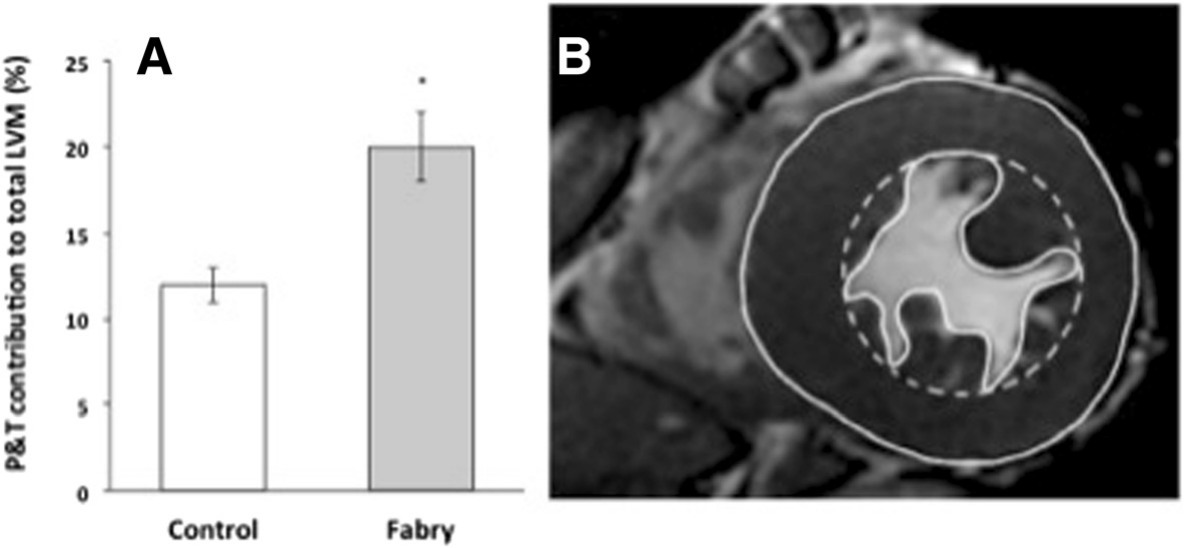
**Papillary muscles and trabecular contributions to total LVM. (A)** Comparison in control versus Fabry subjects (p = 0.01). **(B)** Graphic showing a mid-ventricular slice illustrating the papillary and trabecular components excluded from the LVM using the M_ex_P&T approach (dashed endocardial contour) compared with their inclusion via the MP&T approach (solid endocardial contour) in a Fabry subject. LVM = left ventricular mass.

By including P&T in the analysis approach (M_*inc*_P&T), the difference in LVM between Fabry subjects and controls subjects was 178 ± 14 vs 130 ± 6 g (p = 0.003). This pathological difference was less evident when P&T were excluded (M_*ex*_P&T: 144 ± 12 vs 113 ± 6 g, p = 0.03). This proportional difference appears to be explained by the fact that the papillary muscles and trabeculations contributed to a greater percentage of cardiac mass in Fabry patients than control subjects (20% ± 1 vs 13 ± 2%, p = 0.01; Figure 3A). Figure 3B is an illustration of the relative contribution of the papillary and trabecular volumes to the overall mass in a Fabry patient.

**Figure 4:**
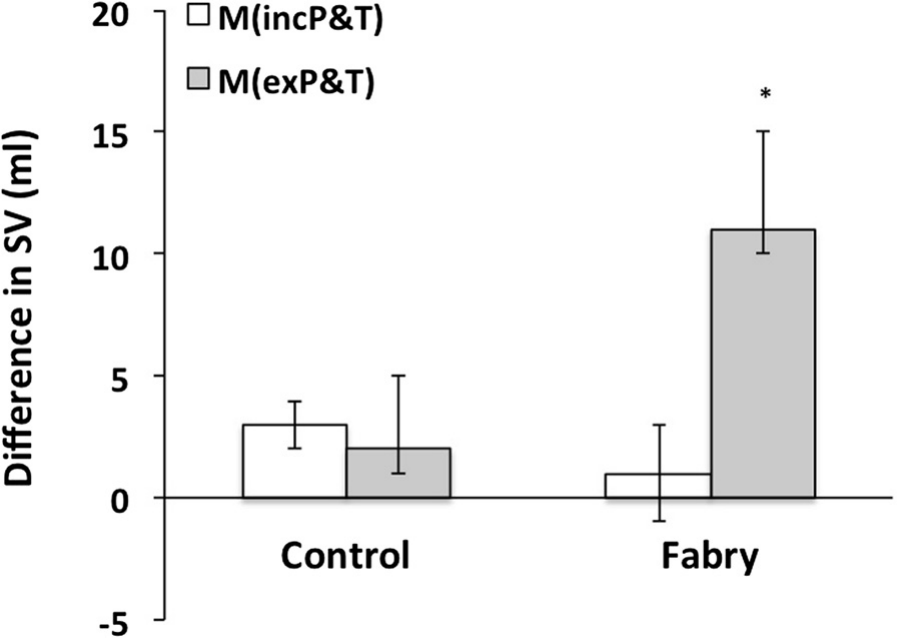
**Effect of volumetric analysis method on the determination of LVSV in control versus Fabry subjects.** Aortic stroke volume, as measured by velocity-encoded flow sequences in the ascending aorta, was subtracted from LVSV, measured using volumetric methods with both M_inc_P&T or M_ex_P&T approaches. LVSV = left ventricular stroke volume.

In order to examine which of the two techniques was more accurate, we compared the stroke volume derived from ventricular contour measurements using both methods with the stroke volume as measured by velocity-encoded flow sequences in the ascending aorta, which is renowned for its accuracy [10,11]. Results are shown in Figure 4. In the control group, both volumetric methods (M_*inc*_P&T or M_*ex*_P&T) gave similar values to the stroke volume calculated from the velocity-encoded data (p = ns). However, in the Fabry group, exclusion of P&T (M_*ex*_P&T) resulted in a significant 12% overestimation of SV compared to aortic flow technique (overestimation of 11 ± 4 ml; p = 0.007). This was corrected by the inclusion of P&T (difference between flow and volume techniques: 1 ± 3 ml, p = 0.7; Figure 4).

### Intra- and inter-observer variability

**Figure 5:**
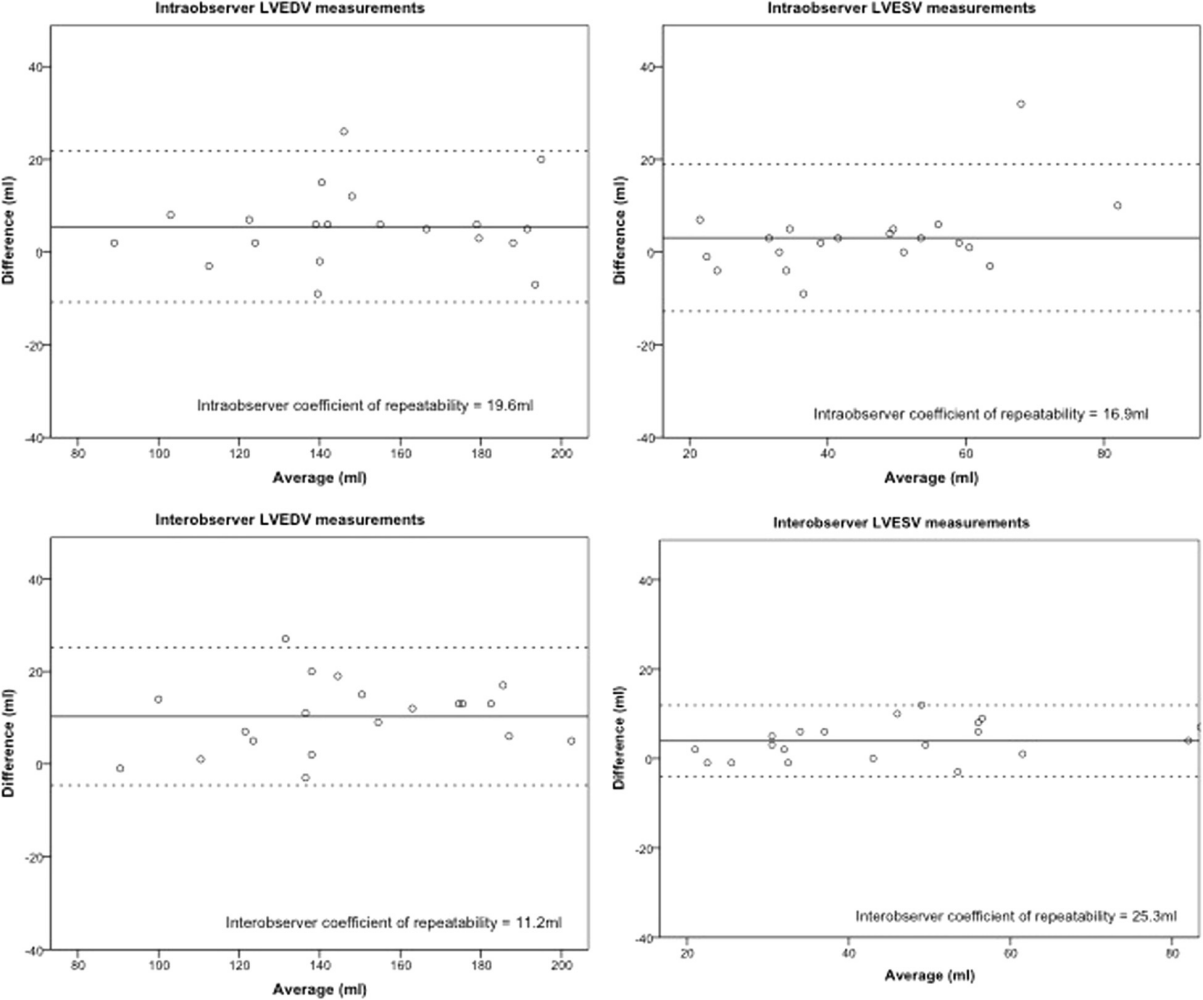
**Bland Altman graphs for LVEDV and LVESV** **(using M**_***inc***_ **P&T** **)** **intraobserver repeatability** (**top panels**) **and interobserver repeatability** **(bottom panels).** The mean difference of each plot is shown as a solid line and the upper and lower limits of agreement as dashed lines. The coefficient of repeatability for each analysis is displayed.

Assessment of the intra- and inter-observer variability showed acceptable levels of agreement for both M_*inc*_P&T and M_*ex*_P&T , using LVEDV and LVESV as example calculations. Figure 5 shows the Bland Altman graphs and coefficients of repeatability.

## Discussion

In this study, we demonstrate that the choice of including or excluding papillary muscles and trabeculations in LV mass measures can result in large differences in the quantification of LV function and mass that is exacerbated in a condition classically associated with cardiac hypertrophy. We show that in patients with Fabry disease, in which sphingolipids are deposited in myocardial tissue, the contribution of papillary and trabecular muscle to the total LV mass is markedly increased compared to healthy controls. The inclusion of papillary muscles in myocardial volume improves accuracy of volumetric measures of LV stroke volume when compared with the internal reference measure taken from velocity encoded flow data at the aortic outflow. These findings may have significant implications for assessment and targeted therapy of patients with Fabry disease, as well as other conditions associated with cardiac hypertrophy, and should prompt a move toward more consistent standards in reporting volumetric data.

Our data demonstrate significant differences between the two analysis approaches for both the Fabry and control groups across a range of parameters. While the impact of analysis approach was modest in the control subjects, the magnitude of these differences was much greater in the Fabry cohort (between 10-30% - see Figure 2). The comparison between the flow-derived LVSV provides strong evidence that M_*inc*_P&T is a more accurate approach in the setting of Fabry-related hypertrophy (Figure 4), and even though for control subjects the comparison was non-significant, it is likely that using the M_*ex*_P&T approach would introduce a bias toward higher LVEDV, LVESV and LVSV with *increasing* hypertrophy. In a population setting, this would have the effect of *over reporting* in these three parameters. We also know, from the Bland Altman plots, that M_inc_P&T is reproducible and reliable.

Recent data using a computer-aided contouring approach in a large sample (n = 1494) drawn from the Framingham study also supports the use of a M_*inc*_P&T approach, demonstrating that the inclusion of P&T in the calculation of LVM results in a measureable difference in normal subjects [12]. The historic motivation for using M_*ex*_P&T contouring was primarily pragmatic – motivated by the relative speed and reproducibility of this technique in normal subjects using manual contouring. Early computer-based contouring routines were not capable of accurate delineation of trabecular contours, hence the use of this technique has been limited by pragmatic considerations [13]. However, in the setting of hypertrophy, the M_*ex*_P&T approach has difficulties in delineating the interface between tightly packed trabeculae and compacted myocardium without careful attention to detail and close reference to the moving cine images [6]. This is especially difficult towards the apex [12]. This technique is therefore not suited to automation in the presence of hypertrophy due to the increase in compacted trabeculations. Although we use a manual approach in this paper, the increasing availability of computer-aided analysis capable of accurate M_*inc*_P&T contours means that the practical advantage of M_*ex*_P&T is no longer compelling.

In the context of cardiac hypertrophy, the effect of type of volumetric analysis approach on the estimation of cardiac mass is of even greater importance than the functional measures. In the Fabry group, papillary muscle and trabecular mass contributed to an average 20% of the total cardiac mass, over one and a half times more than that of the control group (Figure 3). Thus, comparing to the normal range calculated using a papillary exclusion method (as recommended by different organisations) will not fully account for this, and will therefore underestimate the degree of hypertrophy and cardiac involvement for a patient with Fabry disease. The distortion of this important measure may have implications for patients’ disease severity classification and therapy management, which at our institutions is based on organ involvement and the Mainz Severity Scale Index (MSSI) scoring system [14]. Currently, the cardiovascular score in the MSSI includes an assessment of “cardiac muscle thickness” based on electrocardiogram and echocardiography features. In this study, the inclusion of P&T in LVM measurements increased the number of Fabry patients exceeding the upper limit of normal from 6/20 to 11/20 after normalization by BSA [15]. Therefore, an additional five Fabry patients were reclassified as having an abnormally high cardiac mass using the M_*inc*_P&T approach. This effect is driven by the increased contribution papillary muscle hypertrophy makes to overall cardiac mass in Fabry disease, versus non-deposition forms of LV hypertrophy, and has important implications for disease severity classification and therapy in this disease.

Only males were analysed in this study to avoid any influence of gender on hypertrophy in general. We acknowledge that this is a limitation considering Fabry disease affects both genders. It is unclear whether the results can be translated to females, given the gender differences in Fabry cardiomyopathy regarding LVH [16].

The findings of our study have considerable importance in the setting of Fabry disease due to the availability of enzyme replacement therapy (ERT) and the targeting of this therapy to those with identified end-organ involvement. ERT has been available since 2001, and data over the last decade suggests that ERT has the potential to change the prognosis of Fabry disease [17-21]. Despite this, there remain numerous uncertainties regarding these agents – these relate to the optimal stage for treatment initiation, considerable heterogeneity of response, and the magnitude of the long-term clinical benefits. The current criteria for initiation of ERT are variable by location, but in Australia include left ventricular hypertrophy as measured by echocardiography or cardiac MRI, or significant arrhythmia. Making use of the M_*inc*_P&T approach in this cohort may have altered the eligibility for ERT for five of this study’s patients. Our data strongly suggest that accurate measurement of LVM is best achieved using a method that accurately defines the endocardial border so as to include all significant trabeculations and papillary muscles. This analysis deliberately focused on examining subjects with Fabry disease versus controls; further studies are required to explore if the results apply to other types of cardiac hypertrophy.

## Conclusion

The volumetric contribution of papillary muscles and trabeculations in Fabry disease is markedly increased relative to healthy controls in males. Failure to account for this results in significant underestimation of LVM and results in misclassification of a proportion of subjects. While Fabry patients represent an uncommon example of hypertrophy, they underline the importance of accurately contouring the endocardial border for quantification of left ventricular assessment.
